# vissE.cloud: a webserver to visualise higher order molecular phenotypes from enrichment analysis

**DOI:** 10.1093/nar/gkad337

**Published:** 2023-05-09

**Authors:** Ahmed Mohamed, Dharmesh D Bhuva, Sam Lee, Ning Liu, Chin Wee Tan, Melissa J Davis

**Affiliations:** Bioinformatics Division, Walter and Eliza Hall Institute of Medical Research, Melbourne, VIC 3052, Australia; Department of Medical Biology, Faculty of Medicine, Dentistry and Health Sciences, University of Melbourne, Parkville, VIC 3010, Australia; Colonial Foundation Healthy Ageing Centre, Walter and Eliza Hall Institute of Medical Research, Melbourne, VIC 3052, Australia; Bioinformatics Division, Walter and Eliza Hall Institute of Medical Research, Melbourne, VIC 3052, Australia; Department of Medical Biology, Faculty of Medicine, Dentistry and Health Sciences, University of Melbourne, Parkville, VIC 3010, Australia; South Australian immunoGENomics Cancer Institute (SAiGENCI), Faculty of Health and Medical Sciences, The University of Adelaide, Adelaide, SA 5005, Australia; Bioinformatics Division, Walter and Eliza Hall Institute of Medical Research, Melbourne, VIC 3052, Australia; Department of Medical Biology, Faculty of Medicine, Dentistry and Health Sciences, University of Melbourne, Parkville, VIC 3010, Australia; Bioinformatics Division, Walter and Eliza Hall Institute of Medical Research, Melbourne, VIC 3052, Australia; Department of Medical Biology, Faculty of Medicine, Dentistry and Health Sciences, University of Melbourne, Parkville, VIC 3010, Australia; South Australian immunoGENomics Cancer Institute (SAiGENCI), Faculty of Health and Medical Sciences, The University of Adelaide, Adelaide, SA 5005, Australia; Bioinformatics Division, Walter and Eliza Hall Institute of Medical Research, Melbourne, VIC 3052, Australia; Department of Medical Biology, Faculty of Medicine, Dentistry and Health Sciences, University of Melbourne, Parkville, VIC 3010, Australia; Frazer Institute, Faculty of Medicine, The University of Queensland, Brisbane, QLD 4102, Australia; Bioinformatics Division, Walter and Eliza Hall Institute of Medical Research, Melbourne, VIC 3052, Australia; Department of Medical Biology, Faculty of Medicine, Dentistry and Health Sciences, University of Melbourne, Parkville, VIC 3010, Australia; South Australian immunoGENomics Cancer Institute (SAiGENCI), Faculty of Health and Medical Sciences, The University of Adelaide, Adelaide, SA 5005, Australia; Frazer Institute, Faculty of Medicine, The University of Queensland, Brisbane, QLD 4102, Australia; Department of Clinical Pathology, Faculty of Medicine, Dentistry and Health Sciences, University of Melbourne, Parkville, VIC 3010, Australia

## Abstract

Gene-set analysis (GSA) dominates the functional interpretation of omics data and downstream hypothesis generation. Despite its ability to summarise thousands of measurements into semantically interpretable components, GSA often results in hundreds of significantly enriched gene-sets. However, summarisation and effective visualisation of GSA results to facilitate hypothesis generation is still lacking. While some webservers provide gene-set visualization tools, there is still a need for tools that can effectively summarize and guide exploration of GSA results. To enable versatility, webservers accept gene lists as input, however, none provide end-to-end solutions for emerging data types such as single-cell and spatial omics. Here, we present vissE.Cloud, a webserver for end-to-end gene-set analysis, offering gene-set summarisation and highly interactive visualisation. vissE.Cloud uses algorithms from our earlier R package vissE to summarise GSA results by identifying biological themes. We maintain versatility by allowing analysis of gene lists, as well as, analysis of raw single-cell and spatial omics data, including CosMx and Xenium data, making vissE.Cloud the first webserver to provide end-to-end gene-set analysis of sub-cellular localised spatial data. Structuring the results hierarchically allows swift interactive investigations of results at the gene, gene-set, and clusters level. vissE.Cloud is freely available at https://www.vissE.Cloud.

## Graphical Abstract

**Graphical Abstract:**
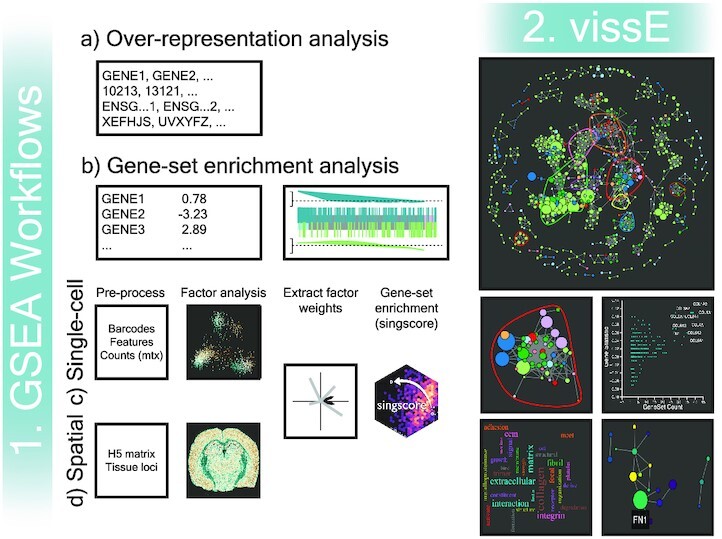
Overview of vissE.Cloud

## INTRODUCTION

High throughput molecular technologies, such as RNA-seq, single-cell RNA-seq, spatial transcriptomics and proteomics, have unlocked new avenues of modelling and understanding the complexity of biological systems. However, this empowerment is dependent on appropriate analysis and interpretation of large high-dimensional data. Many statistical and computational approaches have been developed to address the challenge of genes/proteins prioritisation based on some statistics (1–3); however, these lists are not easily interpretable biologically. Gene-set analysis (GSA) approaches have been developed to address the problem of biological interpretation (4). These approaches use existing functional knowledgebases such as Gene Ontology (5,6), Kyoto Encyclopedia of Genes and Genomes (KEGG) (7) and Reactome pathways (8) that are commonly represented as sets of genes to infer biological functions by assessing the enrichment of prioritised genes.

Though GSA can summarise thousands of measurements into semantically interpretable components, it still presents experimental researchers with two major challenges. First, GSA often results in hundreds of significantly enriched gene-sets, primarily due to redundancy within and between knowledgebases (9–11). Since not all of these hypotheses can be tested, experimental researchers are faced with the decision to prioritise a subset to further follow-up, however tools that facilitate navigating through different hypotheses still lacking. Second, the application of GSA to new technologies such as single-cell and spatial omics requires specialized analytical approaches that can account for the unique features of these data (12–15). Deployment of such rapidly evolving approaches in a scalable manner to web-based applications remains challenging because of the computational and software engineering requirements.

Existing popular GSA web servers attempted to partially address the issues of result summarisation and broad applicability (Table 1) (16–25). For example, WebGestalt (18), g:profiler (22) and Enrichr (19) provide gene-set visualisation tools, however, tools to summarise and explore the results in a guided manner are still lacking. Additionally, web servers provide a limited set of methods, often only over-representation analysis (ORA) and gene-set enrichment analysis (GSEA) (26). To enable versatility of analyses, they require gene lists or ranked gene lists as input. However, they do not provide end-to-end solutions for the emerging data types such as single-cell and spatial omics.

**Table 1.** Comparison of functionality in gene-set analysis web servers. Filled boxes indicate the web server caters for the specific function, while blank boxes indicate lack thereof. Coloured bars label different aspects of the analysis, such as support of different input types, available gene-set databases and representation and visualisation of gene-set analysis results

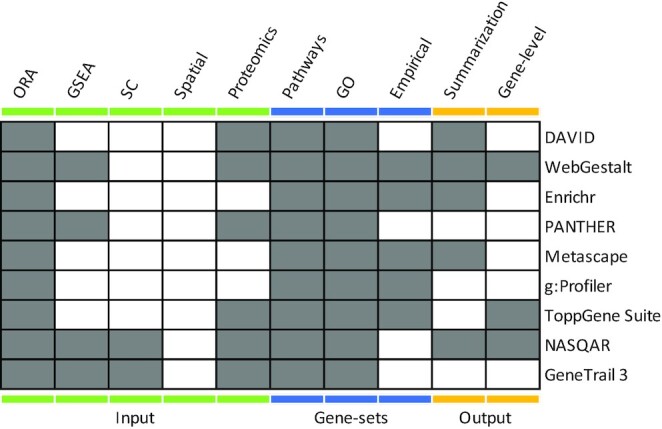

We have previously addressed the issue of result summarisation of GSA results in our R/Bioconductor package, vissE (27), where we identify gene-set clusters with common biological themes. To further empower biologists/scientists with limited coding experience and to enhance result interpretation with interactive visualisations, we present vissE.Cloud. vissE.Cloud provides end-to-end gene-set analysis and offers within-browser gene-set summarisation and highly interactive visualisations. Built upon a job queuing architecture, an R-based analysis core and a Single-Page App frontend, vissE.cloud offers a robust and easily scalable solution for running computationally intensive workflows while providing a streamlined deployment of rapidly evolving methodologies for newer omics technologies. vissE.Cloud supports both ORA and GSEA methods unlike many existing webservers. We maintain versatility by allowing analysis with lists of genes, but in addition, we also support the analysis of single-cell and spatial omics data from the raw data, including pre-processing, factor analysis and factor interpretation. Our easily extensible design allows for the workflow to be deployed to the latest sub-cellular spatial molecular technologies such as CosMx (28) and Xenium (29), making vissE.Cloud the first webserver to enable end-to-end gene-set analysis of sub-cellular localised data. Hierarchical structuring of GSA results, coupled with highly interactive visualisations allow biologists to conduct swift interactive investigations of results at multiple levels/scales, including the gene, gene-set and clusters level yet allowing seamless linkage across all three levels. Till this end, this framework enables a holistic interpretation of biological systems that is intuitive, easily accessible, and interactive for any biologist/scientist to use. vissE.Cloud is freely available at https://www.vissE.Cloud.

## MATERIALS AND METHODS

### Overview of vissE.cloud workflow

**Figure 1.**
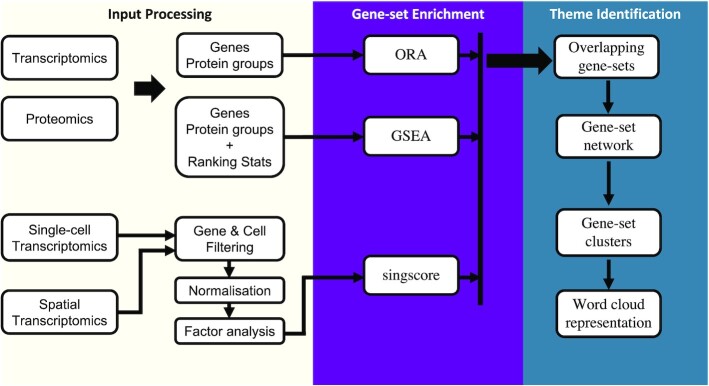
Overall workflow of vissE.Cloud.

The vissE.Clould workflow consists of three main steps (Figure 1): (i) input data processing, (ii) identification of enriched gene-sets and (iii) identification of biological themes/clusters. We describe each of the steps briefly below and refer to the full methodology provided on the help pages via the website.

### Input data processing

To maintain versatility, vissE.Cloud accepts a wide range of inputs allowing the integration of the workflow with differential analysis of bulk transcriptomics, proteomics, single-cell and spatial transcriptomics data.

For bulk transcriptomics, users can choose from two input options: (i) a list of genes of interest, such as those found to be significant in a differential analysis and (ii) genes with their associated statistics, commonly log-fold change or *P*-value. vissE.Cloud supports seven different gene ID types, including UniProt, that are then mapped to their corresponding gene-sets. To facilitate proteomics analysis, vissE.Cloud can handle protein groups commonly produced from proteomics search tools such as MaxQuant (30) and DIA-NN (31).

For single-cell and spatial transcriptomics, vissE.Cloud accepts raw files as input and provides an end-to-end factor analysis and interpretation workflow. Pre-processing of raw data follows the *Orchestrating Single-Cell Analysis* (OSCA) workflow (12), where poor quality cells and lowly variable genes are removed, and data normalised for compositional biases (32), followed by feature extraction of highly variable genes using the scran R package (33). For the panel-based sub-cellular spatial molecular datasets from the CosMx and Xenium technologies, pre-processing follows the pipeline described in (34). Cell-level quality control is performed using spike-in probes that are present in the standard panel. Finally, factor analysis is conducted on log-transformed pre-processed data using principal components analysis (PCA) or non-negative matrix factorisation (NMF) with methods implemented in the scater R package (35). Users are able for fully control the pre-processing parameters from the vissE.Cloud interface.

### Identification of enriched gene-sets

vissE.Cloud compiles gene-sets from Molecular Signatures Database (MSigDB v7.5) (26,36), which includes 31 508 gene-sets split into nine categories and 23 subcategories. This comprehensive compendium of biological knowledge organised into gene-sets is suitable for a wide range of applications, including functional enrichment, regulome analysis, and cell type annotation. Users can select a subset of (sub-)collections depending on the biological hypothesis of interest.

The methodology for identifying enriched gene-sets depends on the user input data. ORA implemented in clusterProfiler (37) is used to identify enriched gene-sets from a list of genes. Alternatively, where a gene-associated statistic is provided, genes are ranked and GSEA is performed using the fgsea R package (38). In both cases, users are able to set *P*-value threshold, or filter gene-set by their size. In factor analysis, gene loadings for each factor are used as gene weights that are subsequently used to score gene-sets using the singscore R package (39,40).

### Identification of biological themes/clusters

The core analysis of biological theme identification is performed using algorithms developed in the vissE R/Bioconductor package (27). Given the results of a GSA analysis, vissE first generates a gene-set network by computing gene-set similarity using the Adjusted Rand Index (ARI) or other user-defined similarity measures. Pairwise similarity represents the number of genes that a pair of gene-sets share or have in common. Gene-set clusters, also referred to as ‘biological themes’, are then identified using a random-walks based graph clustering algorithm (41). These gene-set clusters are ranked based on a combination of the gene-set cluster size and the average of the gene-set statistic for the gene-sets in each cluster. Specifically, a product of ranks statistic (42) is computed using these two metrics such that gene-set clusters having many gene-sets and highly significant gene-sets are prioritised (27).

A semantic meaning is generated for each cluster using natural language processing by performing term frequency analysis on the gene-set names, treating each gene-set name as a document. The term-frequency inverse-document frequency (TF-IDF) is computed for all words within the gene-set cluster. These results are then presented as word clouds with the TF-IDF score determining the size of the word. To link gene-set clusters to their member genes, gene-level statistics are projected on a protein-protein interaction network (43) and are used to generate gene-statistic scatter plots. Details of these methods are described in Bhuva et al. (27).

## RESULTS

### Web server design and architecture

**Figure 2.**
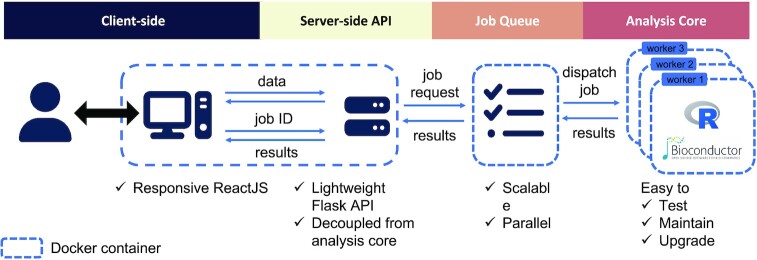
Modular server architecture of vissE.Cloud.

The overall architecture for vissE.Cloud is shown in Figure 2. The client-side is implemented with highly responsive ReactJS. When users submit analysis jobs, they are assigned a human-readable job ID that can be easily shared with collaborators or bookmarked. A python-Flask backend passes all job parameters to a Redis-based job queue where they are dispatched to ‘worker’ processes that performs the analysis in isolated R environments. Finished job results are then formatted as JSON and passed back to the client-side for rendering and visualisation. To achieve separation of concerns and smooth deployment, each of the server components is containerised and the full setup is deployed using docker-compose container orchestration. This modularised structure where modules can be deployed on several compute-instances ensures future scalability for computationally intensive analyses of single-cell and spatial transcriptomics datasets. Currently, the end-to-end analysis of such data takes between five to thirty minutes, providing a quick turnaround time.

### Interactive results visualisation

**Figure 3.**
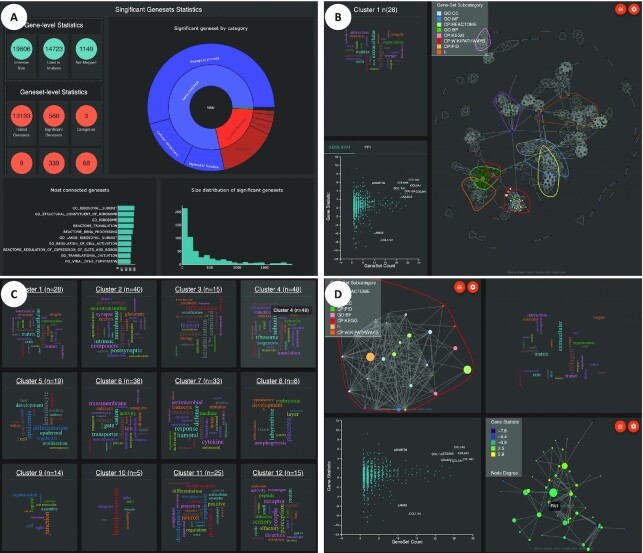
Visualisation view panels in vissE.Cloud. (**A**) GSA overview and summary statistics view. (**B**) Global gene-set network view. (**C**) Cluster gallery view. (**D**) cluster detail view.

vissE.Cloud presents analysis results using three main views that the user can access from the side panel (Figure 3A–C): (i) GSA overview and summary statistics panel, which includes detailed information for the number of mapped genes and tested gene-sets (Figure 3A), (ii) global gene-set network view of the graph along with the identified clusters, associated word cloud and gene/protein statistic plot (Figure 3B) and (iii) cluster gallery view, where identified clusters are represented semantically using word clouds (Figure 3C). An additional detailed cluster view (Figure 3D) is presented where users can select specific themes/clusters to explore depending on the hypotheses of interest. Results are hierarchically structured to allow users to traverse across gene, gene-set and clusters levels seamlessly. We describe each of these views below.

### GSA overview and summary statistics

In this view, important GSA summary statistics associated with genes, gene-sets and clusters are displayed to allow users to validate and identify any issues that may occur in the GSA step. For example, vissE.Cloud shows the number of genes that were mapped to gene-sets in the database used, and that subsequently contributed to the GSA results. This information can reveal mismatching gene identifiers as a useful quality control step. At the gene-set level, distribution of their sizes and categories can uncover potential analysis bias. Additionally, these results can also inform the feasibility of theme identification: if the number of significant gene-sets is very small (in the 10s), any further result summarisation may not be necessary and may not reveal much more that a classic GSA analysis. Collectively, these summary statistics can guide users to understand the overall known functional information content of their data and the status of the GSA analysis performed.

### Global gene-set network view

Here, users can investigate GSA results holistically by visualising the relationships between all significant gene-sets as a gene-set network, potentially uncovering consistent yet previously unknown patterns across the experiment. Different gene-set statistics and annotations such as (sub-)category, false discovery rate (FDR), gene-set size, enrichment score and node degree (a statistic to represent the connectivity of a gene-set) can be used to annotate the colour and size of nodes interactively. Users can change the colour scheme, choosing from 47 different palettes to use for visualisation. To enhance the user experience, site-wide preferred palettes can be specified for categorical, sequential, and diverging data types. Furthermore, this view offers agile network-based navigation between clusters. Interacting with the network by hover over gene-sets (nodes) highlights the gene-set cluster, shows the corresponding word cloud, and displays gene-level statistics and gene-gene interactions inferred from known protein-protein interaction networks (43).

### Cluster gallery view

Since gene-set clusters are composed of numerous gene-sets, vissE.Cloud semantically describe them as word cloud to facilitate user exploration. Interactively, the ‘WordCloud Gallery’ panel renders word clouds progressively as a continuous feed, allowing optimised visualisation of even hundreds of clusters. The biological terms presented by the word clouds augment the user's domain knowledge of the biological systems of interest, relating the identified terms to the biology. As such, the relatively loose semantic definitions afforded by word clouds can be combined with expert domain knowledge to come up with a more complete semantic interpretation. The ‘WordCloud Gallery’ panel provides users entry into more detailed cluster exploration views provided by vissE.Cloud.

### Cluster details view

This view allows thorough investigation of the composition and semantics of a gene-set cluster. Focusing on a selected cluster, vissE.Cloud displays four panels encompassing information across the three hierarchical levels. These panels are: (i) cluster-level word cloud description, (ii) gene-set level similarity network, (iii) gene-level statistics scatter plot and (iv) protein–protein interaction (PPI) network for the genes in the cluster. Collectively, presenting this multiple hierarchical information as a single inter-linked view greatly enhances the interpretation of results in the context of the biological system being studied.

### Factors summary view (single-cell and spatial transcriptomics)

The results of the factor analysis of single-cell and spatial transcriptomics data performed using PCA are displayed in the ‘Factors Summary’ view. This view presents the top factors as word cloud panels, showing four genes-set clusters of each factor. This word cloud panelled view is coupled with the corresponding dimension reduction plots visualising the selected factor of interest. In the case of single-cell transcriptomics data, uniform manifold approximation and projection (UMAP) (44), t-stochastic neighbour embedding (t-SNE) (45) and PCA projections can be visualised. In each of these factor visualisations, data points (cells/loci) can be coloured with user-defined palettes by one of the following data annotations: (i) the top 5 principal components (PCs), (ii) the first two dimensions of the UMAP, (iii) the first two dimensions of the t-SNE or (iv) quality control statistics such as the library size, percent mitochondrial transcripts, and the total number of genes detected. For Visium data, an additional ‘tissue’ dimension allows visualisation of the data in the context of the tissue location of each spot in the spatial data. If histology images (e.g. hematoxylin and eosin (H&E) stained images) are available for the Visium data, they can be uploaded using this view and used as underlays for the tissue plot. Upon selecting a factor, users can access all of the views mentioned above to explore and characterise the biological functions represented by the factor.

## DISCUSSION

To generate biological hypotheses from ‘omics data, should researchers focus on a handful of important genes or on overall trends and hallmarks? This dilemma of determining the appropriate scale and scope of bioinformatics analysis has been a challenging issue preventing researchers from fully unleashing the potential of high-dimensional and high-resolution molecular data. On the one hand, gene function is dependent on molecular contexts and interactions that are often omitted if hypotheses are solely developed from focusing on a few important genes. On the other hand, it is also important to select a distinct subset of genes that represent observed trends to generate more specific testable hypothesis (e.g. using perturbation models).

The solution presented in vissE.Cloud enables researchers to toggle between very broad trends represented in biological themes or gene-set clusters, and narrower contexts at the gene level. Empowered by highly interactive visualisations, researchers can generate hypotheses using both top-down and bottom-up approaches. In the top-down approach, users start with a biological theme of interest and investigate changes in their member gene-sets and genes. Alternatively, users can start with genes of interest and explore how their expression contribute to observed overall trends.

By having an analysis core based on the R framework ensures vissE.Cloud remains versatile and abreast with newly developed methods. Supporting different input types including proteomics, single-cell, and spatial data, vissE.Cloud offers no-code access to methods that would otherwise require high technical expertise. The software engineering challenges of performing R-based long-running analyses while maintaining interactivity were addressed by coupling the backend server with a job queueing service and resolving the architectural complexity with container orchestration. This modular architectural design not only enables rolling out new methods and algorithms for emerging data types but can also be adapted to deploy a wide range of R-based bioinformatics pipelines beyond the scope of vissE.Cloud.

This no-code access and intuitive interactive interface via the cloud will enable a widespread uptake of vissE.Cloud by researchers, even those users with limited bioinformatics experience. The ability to quickly deploy new methods and algorithms will enable high quality research with quality GSA results and visualisations.

## DATA AVAILABILITY

The analysis core for the web server including example data is available as a R package on GitHub at https://github.com/ahmohamed/vissEServerRpkg and on Zenodo at https://doi.org/10.5281/zenodo.7841244. The docker-compose setup for the full architecture is available upon request.
